# Polydeoxyribonucleotide Exerts Therapeutic Effect by Increasing VEGF and Inhibiting Inflammatory Cytokines in Ischemic Colitis Rats

**DOI:** 10.1155/2020/2169083

**Published:** 2020-02-21

**Authors:** Sung-Eun Kim, Il-Gyu Ko, Jun-Jang Jin, Lakkyong Hwang, Chang-Ju Kim, Sang-Hoon Kim, Jin-Hee Han, Jung Won Jeon

**Affiliations:** ^1^Department of Physiology, College of Medicine, Kyung Hee University, Seoul, Republic of Korea; ^2^Department of Anesthesiology and Pain Medicine, Kyung Hee Medical Center, College of Medicine, Kyung Hee University, Seoul, Republic of Korea; ^3^Department of Internal Medicine, Kyung Hee University Hospital at Gangdong, College of Medicine, Kyung Hee University, Seoul, Republic of Korea

## Abstract

Ischemic colitis is resulted from an inadequate blood supply to a segment or entire colon. Polydeoxyribonucleotide (PDRN), extracted from salmon sperm, has been reported to exert anti-inflammatory and anti-ischemic effects through the adenosine A_2A_ receptor (A_2A_R). We investigated whether PDRN possesses therapeutic effectiveness on ischemic colitis rats. Ischemic colitis was induced by selective devascularization. The skin temperature on the ischemic colitis-induced region was determined. To assess the colonic damage score and collagen deposition, colonic tissue sections were stained with hematoxylin and eosin (H&E), and Masson trichrome staining was performed. Western blot analysis for A_2A_R, vascular endothelial growth factor (VEGF), cyclooxygenase-2 (COX-2), tumor necrosis factor-*α* (TNF-*α*), interleukin-1*β* (IL-1*β*), and IL-6, Bax, Bcl-2, and extracellular signal-regulated kinase 1/2 (ERK1/2) was performed. Skin temperature was increased and mucosal damage and collagen deposition were observed in the affected colonic tissues in the ischemic colitis rats. Expressions of inflammatory cytokines (TNF-*α*, IL-1*β*, and IL-6) and inflammatory mediator (COX-2) were upregulated in the ischemic colitis rats. Apoptosis was increased by decreasing the ratio of Bcl-2 to Bax and by suppressing the phosphorylated form of ERK1/2 expression in the ischemic colitis rats. Treatment with PDRN alleviated mucosal damage reduced the expressions of inflammatory cytokines and COX-2 and inhibited apoptosis in the ischemic colitis rats. PDRN treatment more enhanced the expressions of A_2A_R and VEGF in the ischemic colitis rats. PDRN showed therapeutic effectiveness on ischemic colitis by increasing VEGF expression and inhibiting inflammatory cytokines and COX-2 through enhancing A_2A_R expression.

## 1. Introduction

Ischemic colitis, the most common ischemic pathology of the gastrointestinal tract, generally results from an inadequate blood supply to a segment of the colon or the entire colon and leading to colonic inflammation and necrosis [1]. Although the incidence of ischemic colitis in the general population is likely underestimated because many cases are misdiagnosed as inflammatory bowel disease, the annual incidence is estimated at 4.5 to 44 cases per 100,000 persons [2, 3]. Ischemic colitis frequently occurs with old people and female predominance, but it is not necessarily limited to older people and is more likely to develop ischemic colitis in young people with smoking and hyperuricemia [4].

The clinical treatment for ischemic colitis varies widely depending on the cause and degree of ischemia. Although there are various etiologies of ischemic colitis, which are unidentified in most cases, the important underlying mechanism is ischemia [1]. The colon is susceptible to ischemia due to its anatomical and physiological features, including relatively low blood flow and less-developed microvasculature plexus compared to the rest of the gastrointestinal system [5]. Moreover, in cases of hypotension, compensatory homeostatic mechanisms redirect blood flow to the brain at the expense of the visceral circulation [6]. Although ischemia is regarded as a self-limiting process of acute inflammation, the gut, as the origin and/or target of numerous inflammatory cytokines, plays an important role in local injury, and it is involved in systemic inflammation related to multiple-organ dysfunction or failure [7]. Likewise, during the onset of ischemic colitis, various factors promote inflammation of colonic endothelial cells, which increase tissue damage [8]. In particular, colonic and intestinal endothelial cell environment possesses both isoforms of cyclooxygenase (COX), and their induction has been demonstrated to occur in response to different inflammatory cytokines, such as interleukin-1*α* (IL-1*α*), IL-1*β*, and tumor necrosis factor-*α* (TNF-*α*). COX-2 is induced by inflammatory stimuli and synthesizes prostaglandins, which mediate the inflammatory process and tissue damage [9, 10].

Angiogenesis is a pivotal process in all types of wound healing, including gastric and colonic mucosal healing [11, 12]. It is regulated by many proangiogenic factors, such as vascular endothelial growth factor (VEGF), fibroblast growth factor, and endothelial growth factor. Of the many growth factors, VEGF is the most potent stimulus for angiogenesis and it plays an important role in colonic mucosal healing [11].

Apoptosis is controlled by the intrinsic pathway or extrinsic pathway or both. The extrinsic pathway is activated by ligand-induced cell surface receptors, such as tumor necrosis factor receptor (TNFR) and Fas. The intrinsic mitochondrial apoptosis pathway is induced by activation of apoptosis-related proteins, including Bax and Bcl-2, or other signaling pathways, including the mitogen-activated protein kinase (MAPK), phospholipase C*γ*-1 (PLC-*γ*1), and phosphatidylinositol 3-kinase (PI3K)/protein kinase B (Akt) pathways [13]. Extracellular signal-regulated kinase 1/2 (ERK1/2) is one of the MAPK families and modulates cell growth, differentiation, and survival [14, 15]. Apoptosis in intestinal epithelial cells is implicated in the occurrence and procession of inflammatory bowel diseases [16].

Polydeoxyribonucleotide (PDRN) is a mixture of nucleotides with molecular weights of 50–1500 kDa that originates from the human placenta or salmon sperm [17]. PDRN facilitates skin regeneration by activating adenosine A_2A_ receptor (A_2A_R) [17]. A_2A_R is involved in the anti-inflammatory pathway and in the VEGF production [17, 18]. PDRN inhibits apoptosis and exerts anti-inflammatory effect by suppressing the production of inflammatory cytokines [12, 18].

Thus, enhancing VEGF production and suppressing inflammation are the therapeutic strategies for the treatment of ischemic colitis. In the present study, we investigated whether PDRN possesses therapeutic effectiveness on ischemic colitis rats.

## 2. Materials and Methods

### 2.1. Experimental Animals

Seven-week-old male Sprague Dawley rats weighing 180 ± 5 g (*n* = 50) were used in this study. All experimental procedures were performed in accordance with the Guidelines for the Care and Use of Animals of the National Institutes of Health (NIH). This study was approved by the Institutional Animal Care and Use Committee of Kyung Hee University (KHUASP[SE]-14–038). Experimental animals were randomly divided into 5 groups (*n* = 10 in each group): sham-operated group, ischemic colitis-induced group, ischemic colitis-induced and 4 mg/kg PDRN-treated group, ischemic colitis-induced and 8 mg/kg PDRN-treated group, and ischemic colitis-induced and 16 mg/kg PDRN-treated group. Animals were provided food and water *ad libitum* and maintained at 24 ± 2°C with 60% humidity under a 12 h light/dark photoperiod (lights on at 7:00 a.m.).

### 2.2. Induction of Ischemic Colitis and Treatments

Ischemic colitis was induced by selective devascularization, using a modified technique described by Irkorucu et al. [19]. The rats were anesthetized with Zoletil 50® (10 mg/kg, i.p.; Virbac Laboratories, Carros, France) and laparotomized. Subsequently, the rats underwent selective devascularization of a 4 cm segment of the descending colon by marginal vessel ligation at 4 points, with all of the vasa recta in between. The midline incision was sutured, and the rats were returned to their cages with free access to food and water for recovery following surgery. A sham laparotomy was performed on rats in the sham-operated group.

The rats were treated with PDRN (Rejuvenex™; Pharma Research Products, Seongnam, Korea) 48 h after induction of ischemic colitis. The timing of PDRN treatment in this study was performed considering the clinical and pathological progress of ischemic colitis [6, 20]. PDRN diluted in 0.9% saline (500 *µ*L) was injected intraperitoneally at respective dose (4, 8, and 16 mg/kg of body weight) daily for 21 consecutive days. To confirm the involvement of adenosine A_2A_ receptor in PDRN, 16 mg/kg 3,7-dimethyl-1-propargylxanthine (DMPX; Sigma Chemical Co., St. Louis, MO, USA) was applied. The rats in the sham-operated group and in the ischemic colitis-induced group received an equivalent volume of 0.9% saline intraperitoneally for the same duration.

### 2.3. Measurement of Skin Temperature on the Ischemic Colitis-Induced Region

The skin temperature on the ischemic colitis-induced region was determined weekly using an infrared digital thermometer (MT6; Raytek Co., Santa Cruz, CA, USA).

### 2.4. Macroscopic Analysis and Tissue Preparation

Immediately before sacrifice, the rats were anesthetized with an intraperitoneal injection of Zoletil 50® (10 mg/kg), and the laparotomy was reperformed. For macroscopic evaluation, the devascularized segment of the colon was exposed and photographed at the same angle and distance. Morphological damage score was evaluated with the Wallace macroscopic score (Table 1) [21].

Immediately after photographing, the rats were sacrificed, and the colon was excised. Excised colonic tissues were immediately frozen at −80°C for western blot analysis and were fixed with a freshly prepared solution of 4% paraformaldehyde in 100 mM phosphate buffer (pH 7.4) for 24 h for histological staining. Following dehydration with 70, 80, 90, and 100% ethanol, the colonic tissue samples were embedded in paraffin and sectioned (5 *µ*m thickness) using a paraffin microtome (Shandon Finesse 325; Thermo Electron Corp., Somerset, NJ, USA). The paraffin-embedded colonic tissue sections were mounted onto gelatin-coated slides and dried in an oven at 37°C overnight.

### 2.5. Hematoxylin and Eosin (H&E) Staining

For morphologic examination, the colonic tissue sections were stained with hematoxylin and eosin (H&E) and evaluated under a light microscope (Olympus, Tokyo, Japan), according to the previously described method [15, 16]. The sections were stained with Mayer's hematoxylin (Sigma-Aldrich, St. Louis, MO, USA) for 30 sec, washed, and stained with eosin (Sigma-Aldrich) for an additional 3 sec. Following dehydration, the slides were mounted using Permount® (Fisher Scientific, Fair Lawn, NJ, USA). Ischemic colonic damage was evaluated with the Wallace microscopic score (Table 1) [21].

### 2.6. Masson Trichrome Staining

To detect collagen fibers in colonic tissues, Masson trichrome staining was performed, according to the previously described method [22]. The sections were deparaffinized and rehydrated with xylene and 100, 95, and 70% ethanol. After washing, the sections were fixed with Bouin's solution (Sigma-Aldrich) for 1 h at 56°C and stained with Weigert's iron hematoxylin (Sigma-Aldrich) for 10 min. After rinsing with running tap water, the sections were stained with Biebrich scarlet-acid fuchsin (Sigma-Aldrich) and then differentiated by incubation in phosphomolybdic-phosphotungstic acid solution (Sigma-Aldrich) for 10–15 min. Without rinsing, the sections were stained with aniline blue solution (Sigma-Aldrich) for 5 min and a light green solution for 1 min, successively. After rinsing, the sections were differentiated in glacial acetic solution for 5 min, dehydrated quickly with 95% ethanol, and cleared with xylene. The slides were mounted with Permount® (Fisher Scientific). After Masson's trichrome staining, the collagen fibers, nuclei, and background were stained blue, black, and red, respectively.

The fibrosis analysis was conducted according to the previously described method [23]. The collagen/area ratio was analyzed also for 3 areas on each slide by Image-Pro® plus computer-assisted image analysis system (Media Cybernetics Inc., Silver Spring, MD, USA) attached to a light microscope (Olympus), and a mean ratio was defined by three investigators separately.

### 2.7. Western Blot Analysis

According to the previously described method [12, 24], western blot analysis was performed. The colonic tissues were lysed in a lysis buffer consisting of 50 mM HEPES (pH 7.5), 150 mM NaCl, 10% glycerol, 1% Triton X-100, 1 mM PMSF, 1 mM EGTA, 1.5 mM MgCl_2_·6 H_2_O, 1 mM sodium orthovanadate, and 100 mM sodium fluoride. The tissue lysate was incubated for 20 min at 4°C and then centrifuged at 14,000 rpm for 30 min.

Protein content was determined using the Bio-Rad colorimetric protein assay kit (Bio-Rad, Hercules, CA, USA). Protein (20 *µ*g) was separated on SDS-polyacrylamide gels and transferred onto nitrocellulose membranes, which were incubated with the following antibodies: rabbit anti-GAPDH (1 : 5,000; AbFrontier, Seoul, Korea), rabbit anti-A_2A_R (1 : 1000; Abcam, Cambridge, MA, USA), mouse anti-Bcl-2 (1 : 1,000; Santa Cruz Biotechnology, Santa Cruz, CA, USA), mouse anti-Bax (1 : 1,000; Santa Cruz Biotechnology), mouse anti-caspase-3 (1 : 1,000; Santa Cruz Biotechnology), goat anti-COX-2 (1 : 1,000; Santa Cruz Biotechnology), goat anti-TNF-*α* (1 : 1,000; Santa Cruz Biotechnology), goat anti-IL-6 (1 : 1,000; Santa Cruz Biotechnology), rabbit anti-IL-1*β* (1 : 1,000; Santa Cruz Biotechnology), mouse anti-VEGF (1 : 1,000; Santa Cruz Biotechnology), rabbit anti-total p44/42 MAPK (ERK1/2) (1 : 1,000, Cell Signaling Technology Inc., Beverly, Massachusetts, USA), and rabbit anti-phospho-p44/42 MAPK (p-ERK1/2) (1 : 1,000, Cell Signaling Technology Inc.). As secondary antibodies, a horseradish peroxidase-conjugated anti-rabbit antibody (1 : 5,000; Vector Laboratories Inc., Burlingame, CA, USA) was used for GAPDH, A_2A_R, IL-1*β*, ERK1/2, and p-ERK1/2. A horseradish peroxidase-conjugated anti-mouse antibody (1 : 5,000; Amersham Pharmacia Biotech GmbH, Freiburg, Germany) was used for Bcl-2, Bax, caspase-3, and VEGF. A horseradish peroxidase-conjugated anti-goat antibody (1 : 10,000; Vector Laboratories Inc.) was used for COX-2, TNF-*α*, and IL-6.

All of the experimental procedures for western blot were performed under standard laboratory conditions and at room temperature, except for the membrane transfer, which was performed at 4°C with a cold pack and prechilled buffer. Bands from the western blot were detected using the enhanced chemiluminescence (ECL) detection kit (Bio-Rad). To compare the relative expression levels of the proteins, the density of the detected bands was measured using Molecular Analyst™, version 1.4.1 (Bio-Rad).

### 2.8. Data Analysis

The data were analyzed with one-way ANOVA and Duncan's *post hoc* test. All values are expressed as the mean ± standard error of the mean (SEM). *P* value less than 0.05 was considered significant.

## 3. Results

### 3.1. Skin Temperature

**Figure 1:**
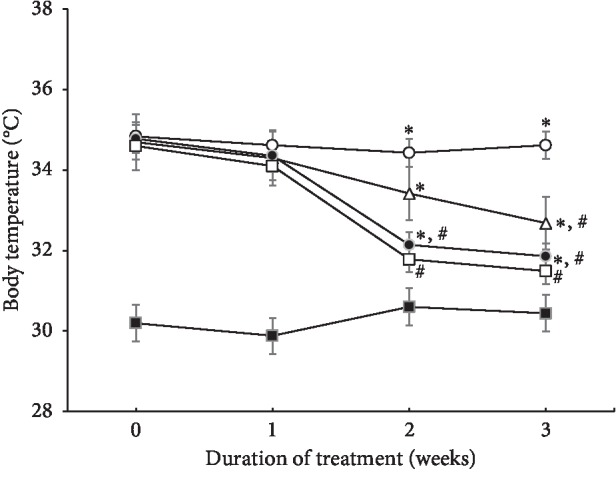
Changes in the skin temperature in ischemic colitis-induced region. (■) The sham-operated group, (○) the ischemic colitis-induced group, (△) the ischemic colitis-induced and 4 mg/kg PDRN-treated group, (●) the ischemic colitis-induced and 8 mg/kg PDRN-treated group, and (□) the ischemic colitis-induced and 16 mg/kg PDRN-treated group. ^*∗*^*P* < 0.05 versus sham-operated group, ^#^*P* < 0.05 versus ischemic colitis-induced group.

Skin temperature on the ischemic colitis-induced region is presented in Figure 1. Induction of ischemic colitis significantly increased the skin temperature of the lesion compared to that in sham rats, and the increased skin temperature was maintained for the first week of treatment with PDRN (*P* < 0.05). However, after 2 weeks, PDRN treatment significantly and dose-dependently decreased the skin temperature of the lesion, and the high dose of PDRN (16 mg/kg) most effectively reduced skin temperature (*P* < 0.05).

### 3.2. Morphological and Histological Changes

**Figure 2:**
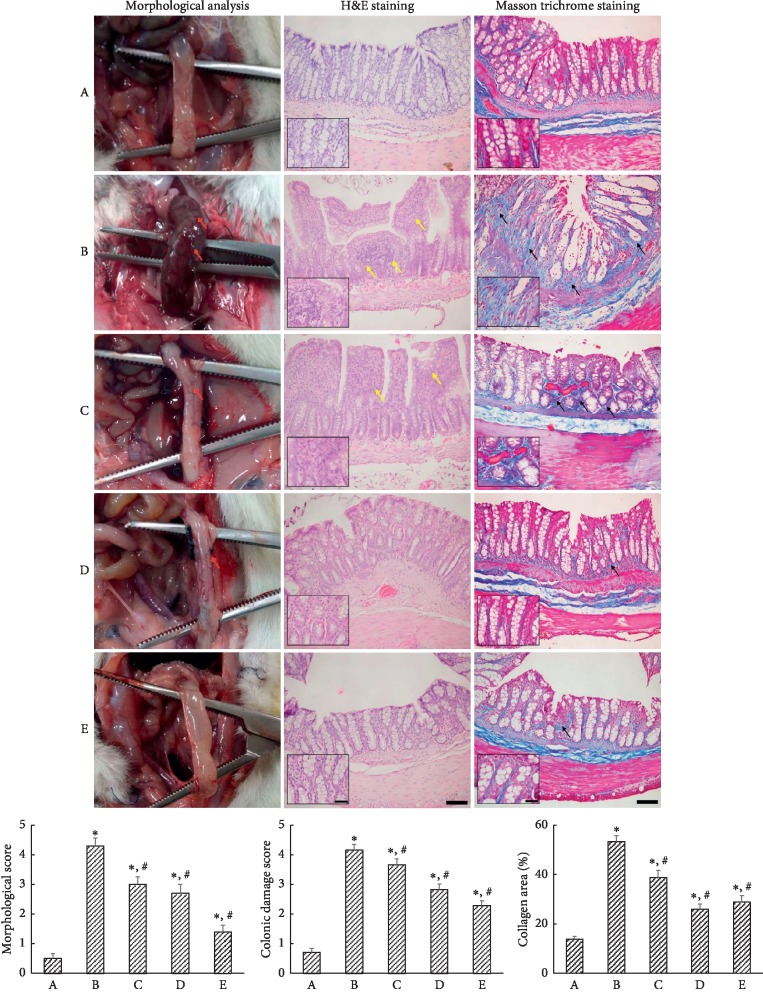
Ischemic colitis-induced morphological and histological changes. Left column: morphological analysis. Red arrows indicate hyperemia and ulceration. Middle column: hematoxylin and eosin staining (nuclei were stained blue and fibers were stained pink). Yellow arrows indicate loss of epithelial cells and distortion of mucosa. Right column: Masson trichrome staining (cytoplasm was stained pink and collagen fibers were stained blue). Black arrows indicate collagen deposition. Lower column: morphological score, colonic damage score, and collagen in each group. (A) The sham-operated group, (B) the ischemic colitis-induced group, (C) the ischemic colitis-induced and 4 mg/kg PDRN-treated group, (D) the ischemic colitis-induced and 8 mg/kg PDRN-treated group, and (E) the ischemic colitis-induced and 16 mg/kg PDRN-treated group. Inset shows histological damage and collagen fibers. The scale bar represents 150 *μ*m (A–E). Insets are higher magnification (scale bar: 50 *μ*m). ^*∗*^*P* < 0.05 versus sham-operated group, ^#^*P* < 0.05 versus ischemic colitis-induced group.

The appearance of the devascularized colon at 3 weeks after induction of ischemic colitis is shown in Figure 2 (left column). Hyperemia and edema were observed in the ischemic colitis-induced rats. PDRN treatment for 3 weeks ameliorated these morphological changes. Colonic mucosa in the sham-operated rats showed a normal appearance with intact epithelium. In contrast, mucosa in the ischemic colitis-induced rats showed mucosal damage with losses of goblet cells and abnormal crypts. PDRN treatment remarkably alleviated the mucosal damage induced by selective devascularization of the colon (Figure 2, middle column). Collagen deposition in the colonic tissue was increased, especially in the mucosal layer, by induction of ischemic colitis. PDRN treatment reduced collagen deposition in the colonic tissue (Figure 2, right column).

These changes led to elevated colonic damage score and collagen (*P* < 0.05). However, PDRN treatment resulted in the dose-dependent decrease in colonic damage score and collagen (*P* < 0.05).

### 3.3. A_2A_R and VEGF Expressions

**Figure 3:**
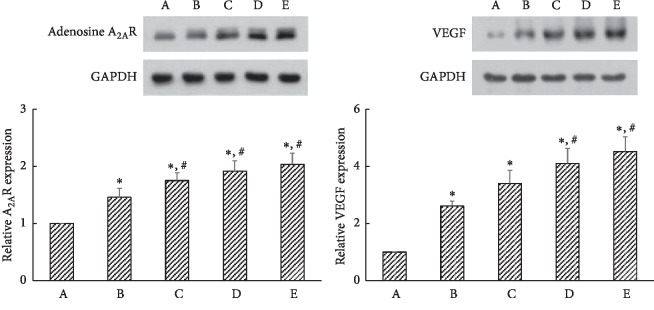
Effects of polydeoxyribonucleotide (PDRN) on the expressions of adenosine A2A receptor (A_2A_R) and vascular endothelial growth factor (VEGF) in ischemic colitis-induced tissues. (A) Sham-operated group, (B) ischemic colitis-induced group, (C) ischemic colitis-induced and 4 mg/kg PDRN-treated group, (D) ischemic colitis-induced and 8 mg/kg PDRN-treated group, and (E) ischemic colitis-induced and 16 mg/kg PDRN-treated group. ^*∗*^*P* < 0.05 versus sham-operated group, ^#^*P* < 0.05 versus ischemic colitis-induced group.

Induction of ischemic colitis increased A_2A_R (45 kDa) and VEGF (21 kDa) expressions in the colonic tissue (*P* < 0.05). Treatment with PDRN further enhanced A_2A_R and VEGF expressions (*P* < 0.05) (Figure 3).

### 3.4. COX-2, TNF-*α*, IL-1*β*, and IL-6 Expressions

**Figure 4:**
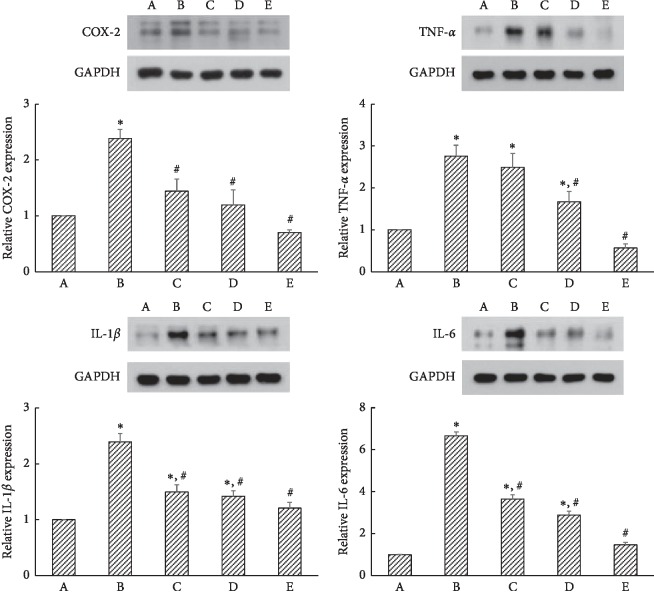
Effects of polydeoxyribonucleotide (PDRN) on the expressions of cyclooxygenase-2 (COX-2), tumor necrosis factor-*α* (TNF-*α*), interleukin-1*β* (IL-1*β*), and IL-6 in ischemic colitis-induced tissues. (A) Sham-operated group, (B) ischemic colitis-induced group, (C) ischemic colitis-induced and 4 mg/kg PDRN-treated group, (D) ischemic colitis-induced and 8 mg/kg PDRN-treated group, and (E) ischemic colitis-induced and 16 mg/kg PDRN-treated group. ^*∗*^*P* < 0.05 versus sham-operated group, ^#^*P* < 0.05 versus ischemic colitis-induced group.

Induction of ischemic colitis increased the expressions of inflammatory proteins, such as COX-2 (70–72 kDa), TNF-*α* (17 kDa), IL-1*β* (17 kDa), and IL-6 (22–27 kDa) in the colonic tissue (*P* < 0.05). In contrast, PDRN treatment dose-dependently reduced COX-2, TNF-*α*, IL-1*β*, and IL-6 expressions in the colonic tissue (*P* < 0.05) (Figure 4).

### 3.5. Bcl-2, Bax, Caspase-3, and Phospho-p44/42 MAPK (p-ERK1/2) Expressions

**Figure 5:**
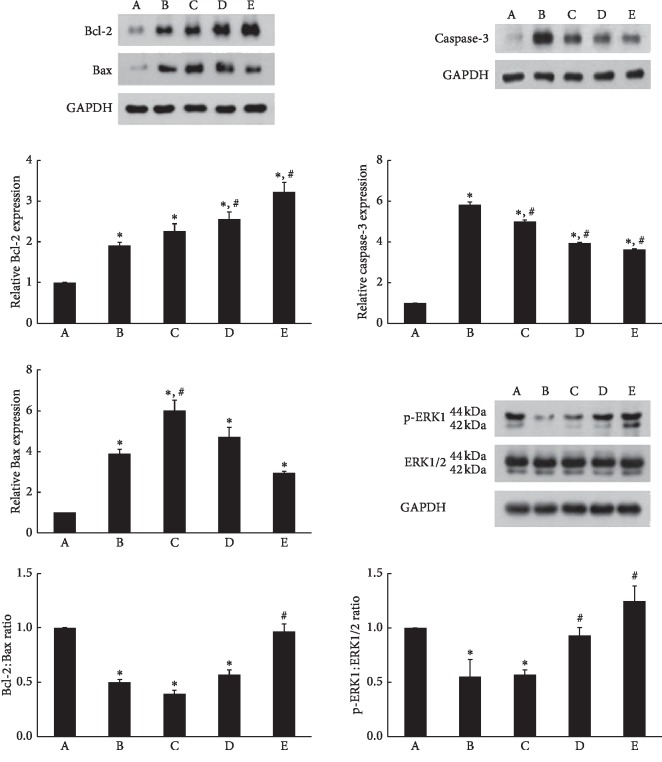
Effects of polydeoxyribonucleotide (PDRN) on the expressions of Bcl-2, Bax, caspase-3, and extracellular signal-regulated kinase 1/2 (ERK1/2) in ischemic colitis-induced tissues. (A) Sham-operated group, (B) ischemic colitis-induced group, (C) ischemic colitis-induced and 4 mg/kg PDRN-treated group, (D) ischemic colitis-induced and 8 mg/kg PDRN-treated group, and (E) ischemic colitis-induced and 16 mg/kg PDRN-treated group. ^*∗*^*P* < 0.05 versus sham-operated group, ^#^*P* < 0.05 versus ischemic colitis-induced group.

Induction of ischemic colitis increased Bax (24 kDa) and Bcl-2 (26–29 kDa) expressions in the colonic tissues. However, the increase of Bax expression was greater than Bcl-2 expression, and consequently, Bcl-2 to Bax ratio was lower in the ischemic colitis-induced rats than in the normal rats (*P* < 0.05). In contrast, treatment with PDRN increased the expression of Bcl-2; as a result, Bcl-2 to Bax ratio was higher in the 16 mg/kg PDRN treatment (*P* < 0.05) (Figure 5, left). Induction of ischemic colitis led to an increase in the caspase-3 (32 kDa) expression (*P* < 0.05). However, PDRN treatment dose-dependently inhibited caspase-3 expression (*P* < 0.05) (Figure 5, right-upper).

Induction of ischemic colitis decreased the expression of phospho-p44/42 MAPK (p-ERK1/2, 42 and 44 kDa) in the colonic tissue when compared to the levels in normal rats (*P* < 0.05). Conversely, PDRN treatment dose-dependently increased the expression of p-ERK1/2 (*P* < 0.05) (Figure 5, right-lower).

### 3.6. Evaluation of Efficacy in PDRN through DMPX

**Figure 6:**
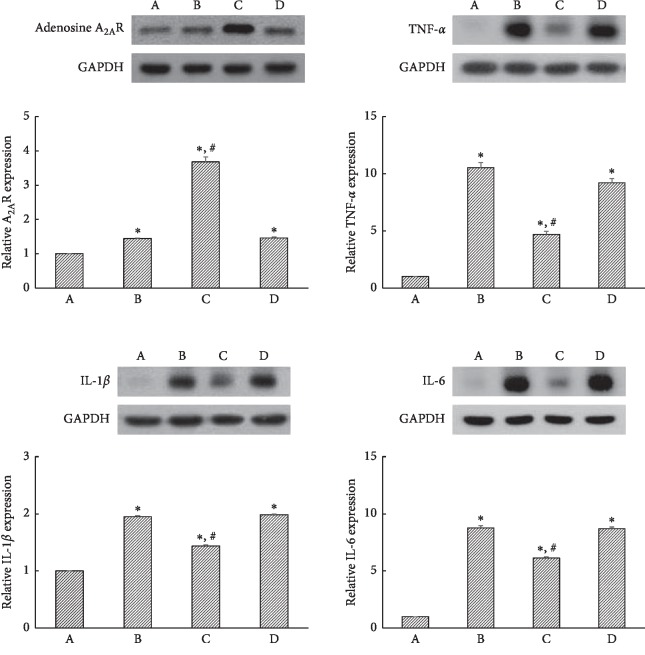
Effects of polydeoxyribonucleotide (PDRN) and 3,7-dimethyl-1-propargylxanthine (DMPX) on the expressions of adenosine A_2A_ receptor (A_2A_R), tumor necrosis factor-*α* (TNF-*α*), interleukin-1*β* (IL-1*β*), and IL-6 in ischemic colitis-induced tissues. (A) Sham-operated group, (B) ischemic colitis-induced group, (C) ischemic colitis-induced and 16 mg/kg PDRN-treated group, and (D) ischemic colitis-induced and 16 mg/kg PDRN + 16 mg/kg DMPX-treated group. ^*∗*^*P* < 0.05 versus sham-operated group, ^#^*P* < 0.05 versus ischemic colitis-induced group.

Induction of ischemic colitis increased TNF-*α*, IL-1*β*, and IL-6 expressions, whereas decreased adenosine A_2A_ receptor expression (*P* < 0.05) (Figure 6). PDRN treatment suppressed expression of TNF-*α*, IL-1*β*, and IL-6, in contrast enhanced the adenosine A_2A_ receptor expression (*P* < 0.05) (Figure 6). In the case of PDRN + DMPX treatment, TNF-*α*, IL-1*β*, and IL-6 levels were not changed compared to ischemic colitis-induced group. Likewise, PDRN + DMPX treatment showed no change in adenosine A_2A_ receptor expression compared to ischemic colitis group.

## 4. Discussion

Inflammatory cytokines, such as TNF-*α*, IL-1*β*, and IL-6, are frequently upregulated in the animal models of experimental colitis [25, 26]. Increased level of TNF-*α* was observed in the serum of patients with ulcerative colitis and Crohn's disease [27, 28]. IL-6 induces TNF-*α* and IL-1*β* production, and IL-6 also increases the expression of adhesion proteins, including intercellular adhesion molecule-1 (ICAM-1), involved in the activation and migration of inflammatory cells to the intestine [29]. IL-6 was elevated during intestinal ischemia and further increased during reperfusion [7]. Increased levels of TNF-*α* and IL-1*β* in the ischemic intestine caused the progress of intestinal injury [30]. As a result, double inhibition of TNF-*α* and IL-1*β* can effectively ameliorate postischemic deterioration of the intestine. PDRN reduced the expressions of inflammatory cytokines and altered the expression of apoptotic markers [26]. Specific COX-2 inhibitors ameliorated the symptoms of inflammation [31]. In the present study, treatment with PDRN effectively suppressed ischemic colitis-induced COX-2, TNF-*α*, IL-1*β*, and IL-6 expressions in the colonic tissues (Figure 4). The present results showed that PDRN potently inhibited inflammation in the ischemic colitis rats.

Adenosine suppresses TNF-*α* release from macrophages, primarily through A_2A_R [32]. Activation of A_2A_R has a robust anti-inflammatory effect, and A_2A_R is involved in the repair of experimental colitis [33, 34]. PDRN potentiated gastric healing by activation of A_2A_R in gastric ulcer rats [24]. In the present study, induction of ischemic colitis increased A_2A_R expression and PDRN treatment further increased A_2A_R expression in colonic tissue (Figure 3, left). The present results showed that the effects of PDRN appeared through the enhancing of A_2A_R expression.

Fibrosis is considered as a consequence of chronic inflammation in inflammatory bowel disease [35]. Theiss et al. [36] suggested that TNF-*α* is associated with intestinal fibrosis, since TNF-*α* induces collagen accumulation and proliferation in intestinal myofibroblasts. Clinically, ischemic colitis is classified as full-thickness transmural colitis and partial-thickness ischemic colitis. The first type is associated with gangrene and multiorgan failure, while the latter is confined to the mucosa and submucosa [1]. In the present study, mucosal damage induced by selective devascularization was observed, which was effectively prevented by PDRN treatment. Administration of PDRN decreased collagen deposition in colonic tissue (Figure 2). The present results showed that PDRN alleviated mucosal damage and decreased collagen deposition in the ischemic colitis rats.

In addition, body temperature is a marker of severity in colitis and inflammation [37], and an increase in skin temperature is an indicator of inflammation. Pallio et al. [26] reported that PDRN improved the features and clinical symptoms of colitis, such as fever, weight loss, and histological alterations. In the present study, PDRN treatment reduced the colitis-induced increase of skin temperature (Figure 1). The present results showed that PDRN relieved the symptoms of ischemic colitis.

Two ischemic factors, VEGF and hypoxia-inducible factor-1*α* (HIF-1*α*), were constitutively expressed in the human colon tissue and overexpressed in the ischemic colitis lesion [38]. In ischemic crises, VEGF and HIF-1*α* were immediately induced and participated in the prevention against the expansion of tissue damage as well as for the repairing damaged tissue [38]. Under ischemic conditions, activation of A_2A_R stimulated VEGF production in macrophages [39]. PDRN facilitated blood flow via stimulation of VEGF in an experimental model of ischemic skin flaps. Increased level of VEGF contributed to angiogenesis and symptom improvement [40, 41]. PDRN maintained high VEGF level during angiogenesis and restoration of blood flow through activation of A_2A_R [18]. Thus, PDRN can be considered to possess anti-ischemic effect [33]. Our study also showed that induction of ischemic colitis increased VEGF expression and PDRN treatment further increased VEGF expression in colonic tissue (Figure 3, right). The present results showed that PDRN facilitated vascularization in the ischemic colitis rats.

Apoptosis was increased in ischemic colitis rats along with inflammation and oxidative damage [8]. Excessive apoptosis damages mucosal integrity and barrier function, leading to mucous epithelial injury, ulcerative formation, inflammatory cell infiltration, and other conditions related to colitis [13, 42]. Pallio et al. [26] reported that PDRN treatment affected Bax and Bcl-2 expressions in experimental colitis by reducing the numbers of apoptotic and necrotic cells in all tissue layers. In their study, TNF-*α* induced either apoptosis or necrosis depending on cell types, environmental conditions, and the magnitude of the cellular insults [26]. Increase of Bcl-2 to Bax ratio represents suppression of apoptosis [43, 44]. In addition, caspase-3, which is the most widely studied caspase, is a key executor of apoptosis [45].

In the present study, induction of ischemic colitis affected the expressions of Bcl-2 and Bax, resulting in decrement of the ratio of antiapoptotic protein Bcl-2 to proapoptotic protein Bax. However, PDRN treatment increased Bcl-2 and suppressed Bax, resulting in enhancement of the ratio of Bcl-2 to Bax (Figure 5, left). Furthermore, our present study showed that PDRN treatment suppressed the ischemic colitis-induced increase in caspase-3 expression (Figure 5, right-upper). The present results showed that PDRN suppressed apoptosis in the ischemic colitis rats.

The protective effect of ERK activation is mediated by antiapoptotic members of the Bcl-2 family, including Bcl-2 and Bcl-xL [14, 15]. Activation of ERK inhibits caspase-8 cleavage and translocation of Bax and consequently suppresses the release of cytochrome *c* from mitochondria [46]. TNF-*α*-induced apoptosis in polyamine-depleted IEC-6 cells, a nontransformed intestinal crypt epithelial cell line, was prevented by ERK1/2 activation [47]. ERK1/2 activation is implicated in the facilitation of regeneration [44]. In the present study, PDRN treatment enhanced phosphorylation of ERK1/2 in the colonic tissues of ischemic colitis rats (Figure 5, right-lower), which might contribute to inhibiting apoptosis. The present results showed that the inhibitory effect of PDRN on apoptosis appeared through phosphorylation of ERK1/2.

## 5. Conclusions

PDRN treatment reduced the morphological score and colonic damage score. This promising therapeutic effect of PDRN on colonic damage may be attributable to its ability to enhance A_2A_R expression. PDRN also improved gastric ulcer healing by suppressing inflammatory cytokines, such as TNF-*α*, IL-1*β*, and IL-6 through A_2A_R. In addition, the present study additionally observed a relationship between DMPX, a specific adenosine A_2A_ receptor antagonist, and PDRN in a model of ischemic colitis. These findings strongly support that PDRN may be efficacious in the treatment. The present results suggest that PDRN possesses therapeutic efficacy for ischemic colitis by increasing VEGF expression and inhibiting inflammatory cytokines through enhancing A_2A_R expression.

## Figures and Tables

**Table 1:** Wallace macroscopic and microscopic colonic damage score.

Score	Appearance
Macroscopic	
0	Normal
1	Local hyperemia without ulceration
2	Ulceration without hyperemia
3	Ulceration with hyperemia at one site
4	Ulceration with hyperemia at two of more sites
5	Ulceration extending more than 2 cm at several sites

Microscopic	
0	Normal
1	Damage limited to surface epithelium
2	Focal ulceration limited to mucosa
3	Focal, transmural inflammation and ulceration
4	Extensive transmural ulceration and inflammation bordered by normal mucosa
5	Extensive transmural ulceration and inflammation involving entire section

## Data Availability

All data generated or analyzed during the present study are included in this article.

## References

[1] Doulberis M., Panagopoulos P., Scherz S., Dellaporta E., Kouklakis G. (2016). Update on ischemic colitis: from etiopathology to treatment including patients of intensive care unit. *Scandinavian Journal of Gastroenterology*.

[2] Scharff J. R., Longo W. E., Vartanian S. M., Jacobs D. L., Bahadursingh A. N., Kaminski D. L. (2003). Ischemic colitis: spectrum of disease and outcome. *Surgery*.

[3] Higgins P. D. R., Davis K. J., Laine L. (2004). The epidemiology of ischaemic colitis. *Alimentary Pharmacology and Therapeutics*.

[4] Kimura T., Shinji A., Horiuchi A. (2012). Clinical characteristics of young-onset ischemic colitis. *Digestive Diseases and Sciences*.

[5] Theodoropoulou Α., Κoutroubakis I. E. (2008). Ischemic colitis: clinical practice in diagnosis and treatment. *World Journal of Gastroenterology*.

[6] Washington C., Carmichael J. C. (2012). Management of ischemic colitis. *Clinics in Colon and Rectal Surgery*.

[7] Lammers K., Innocenti G., Venturi A. (2003). The effect of transient intestinal ischemia on inflammatory parameters. *International Journal of Colorectal Disease*.

[8] Karatepe O., Cakir A., Unal O. (2011). Iloprost reduces colonic injury in ischemic colitis in rats. *Acta Cirurgica Brasileira*.

[9] Caughey G. E., Cleland L. G., Penglis P. S., Gamble J. R., James M. J. (2001). Roles of cyclooxygenase (COX)-1 and COX-2 in prostanoid production by human endothelial cells: selective up-regulation of prostacyclin synthesis by COX-2. *The Journal of Immunology*.

[10] Lugo B., Ford H. R., Grishin A. (2007). Molecular signaling in necrotizing enterocolitis: regulation of intestinal COX-2 expression. *Journal of Pediatric Surgery*.

[11] Sandor Z., Deng X. M., Khomenko T., Tarnawski A. S., Szabo S. (2006). Altered angiogenic balance in ulcerative colitis: a key to impaired healing?. *Biochemical and Biophysical Research Communications*.

[12] Jeon J. W., Lee J. I., Shin H. P. (2014). Adenosine A2A-receptor agonist polydeoxyribonucleotide promotes gastric ulcer healing in Mongolian gerbils. *Animal Cells and Systems*.

[13] Dirisina R., Katzman R. B., Goretsky T. (2011). p53 and PUMA independently regulate apoptosis of intestinal epithelial cells in patients and mice with colitis. *Gastroenterology*.

[14] Deng X., Ruvolo P., Carr B., May W. S. (2000). Survival function of ERK1/2 as IL-3-activated, staurosporine-resistant Bcl2 kinases. *Proceedings of the National Academy of Sciences*.

[15] May M., Uchida M., Watanabe T. (2003). Activation of extracellular signal-regulated kinases ERK1 and ERK2 induces Bcl-xL up-regulation via inhibition of caspase activities in erythropoietin signaling. *Journal of Cellular Physiology*.

[16] Seidelin J. B., Nielsen O. H. (2008). Attenuated apoptosis response to Fas-ligand in active ulcerative colitis. *Inflammatory Bowel Diseases*.

[17] Veronesi F., Dallari D., Sabbioni G., Carubbi C., Martini L., Fini M. (2017). Polydeoxyribonucleotides (PDRNs) from skin to musculoskeletal tissue regeneration via adenosine A2A receptor involvement. *Journal of Cellular Physiology*.

[18] Altavilla D., Bitto A., Polito F. (2009). Polydeoxyribonucleotide (PDRN): a safe approach to induce therapeutic angiogenesis in peripheral artery occlusive disease and in diabetic foot ulcers. *Cardiovascular & Hematological Agents in Medicinal Chemistry*.

[19] Irkorucu O., Taşcilar O., Çakmak G. K. (2008). The effect of sildenafil on an animal model for ischemic colitis. *Digestive Diseases and Sciences*.

[20] Brandt L. J., Feuerstadt P. (2016). Beyond low flow: how I manage ischemic colitis. *American Journal of Gastroenterology*.

[21] Wallace J. L., Keenan C. M. (1990). An orally active inhibitor of leukotriene synthesis accelerates healing in a rat model of colitis. *The American Journal of Physiology*.

[22] Korehisa S., Ikeda T., Okano S. (2018). A novel histological examination with dynamic three-dimensional reconstruction from multiple immunohistochemically stained sections of a PD-L1-positive colon cancer. *Histopathology*.

[23] Zhu M. Y., Lu Y. M., Ou Y. X., Zhang H. Z., Chen W. X. (2012). Dynamic progress of 2,4,6-trinitrobenzene sulfonic acid induced chronic colitis and fibrosis in rat model. *Journal of Digestive Diseases*.

[24] Ko I.-G., Kim S.-E., Jin J.-J. (2018). Combination therapy with polydeoxyribonucleotide and proton pump inhibitor enhances therapeutic effectiveness for gastric ulcer in rats. *Life Sciences*.

[25] Jin H., Guo J., Liu J. (2017). Anti-inflammatory effects and mechanisms of vagal nerve stimulation combined with electroacupuncture in a rodent model of TNBS-induced colitis. *American Journal of Physiology-Gastrointestinal and Liver Physiology*.

[26] Pallio G., Bitto A., Pizzino G. (2016). Adenosine receptor stimulation by polydeoxyribonucleotide improves tissue repair and symptomology in experimental colitis. *Frontiers in Pharmacology*.

[27] Billmeier U., Dieterich W., Neurath M. F., Atreya R. (2016). Molecular mechanism of action of anti-tumor necrosis factor antibodies in inflammatory bowel diseases. *World Journal of Gastroenterology*.

[28] Holtmann M. H., Schütz M., Galle P. R., Neurath M. F. (2002). Functional relevance of soluble TNF-*α*, transmembrane TNF-*α* and TNF-signal transduction in gastrointestinal diseases with special reference to inflammatory bowel diseases. *Zeitschrift für Gastroenterologie*.

[29] Atreya R., Neurath M. F. (2005). Involvement of IL-6 in the pathogenesis of inflammatory bowel disease and colon cancer. *Clinical Reviews in Allergy & Immunology*.

[30] Yamamoto S., Tanabe M., Wakabayashi G., Shimazu M., Matsumoto K., Kitajima M. (2001). The role of tumor necrosis factor-*α* and interleukin-1*β* in ischemia-reperfusion injury of the rat small intestine. *Journal of Surgical Research*.

[31] Crofford L. J., Lipsky P. E., Brooks P., Abramson S. B., Simon L. S., van de Putte L. B. A. (2000). Basic biology and clinical application of specific cyclooxygenase-2 inhibitors. *Arthritis & Rheumatism*.

[32] Kreckler L. M., Wan T. C., Ge Z.-D., Auchampach J. A. (2006). Adenosine inhibits tumor necrosis factor-*α* release from mouse peritoneal macrophages via A_2A_ and A_2B_ but not the A_3_ adenosine receptor. *Journal of Pharmacology and Experimental Therapeutics*.

[33] Squadrito F., Bitto A., Irrera N. (2017). Pharmacological activity and clinical use of PDRN. *Frontiers in Pharmacology*.

[34] Antonioli L., Fornai M., Colucci R. (2010). The blockade of adenosine deaminase ameliorates chronic experimental colitis through the recruitment of adenosine A_2A_ and A_3_ receptors. *Journal of Pharmacology and Experimental Therapeutics*.

[35] Lund K. P., Rigby R. J. (2008). What are the mechanisms of fibrosis in IBD?. *Inflammatory Bowel Diseases*.

[36] Theiss A. L., Simmons J. G., Jobin C., Lund P. K. (2005). Tumor necrosis factor (TNF) *α* increases collagen accumulation and proliferation in intestinal myofibroblasts via TNF receptor 2. *Journal of Biological Chemistry*.

[37] Meregnani J., Clarençon D., Vivier M. (2011). Anti-inflammatory effect of vagus nerve stimulation in a rat model of inflammatory bowel disease. *Autonomic Neuroscience: Basic & Clinical*.

[38] Okuda T., Azuma T., Ohtani M. (2005). Hypoxia-inducible factor 1 alpha and vascular endothelial growth factor overexpression in ischemic colitis. *World Journal of Gastroenterology*.

[39] Ernens I., Léonard F., Vausort M., Rolland-Turner M., Devaux Y., Wagner D. R. (2010). Adenosine up-regulates vascular endothelial growth factor in human macrophages. *Biochemical and Biophysical Research Communications*.

[40] Bitto A., Polito F., Altavilla D., Minutoli L., Migliorato A., Squadrito F. (2008). Polydeoxyribonucleotide (PDRN) restores blood flow in an experimental model of peripheral artery occlusive disease. *Journal of Vascular Surgery*.

[41] Polito F., Bitto A., Galeano M. (2012). Polydeoxyribonucleotide restores blood flow in an experimental model of ischemic skin flaps. *Journal of Vascular Surgery*.

[42] Liu D. Y., Xu R., Huang M. F. (2015). Si Shen Wan regulates phospholipase C*γ*-1 and PI3K/Akt signal in colonic mucosa from rats with colitis. *Evidence-Based Complementary and Alternative Medicine*.

[43] Baek S.-S., Kim S.-H. (2016). Treadmill exercise ameliorates symptoms of Alzheimer disease through suppressing microglial activation-induced apoptosis in rats. *Journal of Exercise Rehabilitation*.

[44] Kim Y.-M., Jin J.-J., Lee S.-J., Seo T.-B., Ji E.-S. (2018). Treadmill exercise with bone marrow stromal cells transplantation facilitates neuroprotective effect through BDNF-ERK1/2 pathway in spinal cord injury rats. *Journal of Exercise Rehabilitation*.

[45] Benchoua A., Guégan C., Couriaud C. (2001). Specific caspase pathways are activated in the two stages of cerebral infarction. *The Journal of Neuroscience*.

[46] Söderström T. S., Poukkula M., Holmström T. H., Heiskanen K. M., Eriksson J. E. (2002). Mitogen-activated protein kinase/extracellular signal-regulated kinase signaling in activated T cells abrogates TRAIL-induced apoptosis upstream of the mitochondrial amplification loop and caspase-8. *The Journal of Immunology*.

[47] Bhattacharya S., Ray R. M., Johnson L. R. (2004). Prevention of TNF-*α*-induced apoptosis in polyamine-depleted IEC-6 cells is mediated through the activation of ERK1/2. *American Journal of Physiology-Gastrointestinal and Liver Physiology*.

